# Workpeople's Subscriptions

**Published:** 1887-10-29

**Authors:** 


### WORKPEOPLE'S SUBSCRIPTIONS.

The following statement is most creditable to the working classes in Sunderland. It shows that through their agency alone nearly sixpence per head of the population is contributed to the hospitals through the workpeople's agency. Northerners are deservedly famous for their manly independence, and this is a striking proof of the fact. We can only hope that the workpeople down south will begin to wake up to a sense of their duty. London, with upwards of five millions of inhabitants, gives less than £10,000 per annum through the Workpeople's Fund and Hospital Saturday; Sunderland, with one-fiftieth of the population, gives £2,469. Once more it may be asked, Can London wait? We think not; we want a proportionate contribution in London, say £120,000 per annum, for the Hospital Saturday Fund. Mr. Webster has done well to call attention to this question:—

"The paragraph in your issue of 8th inst., referring to the increased support given by the workpeople of Leeds to their local infirmary, is misleading. You say, 'The workpeople of no town of the same magnitude contributed anything like the sum of £1,869 17s. 11d. per annum to their local hospital.' The population of Leeds at last census was something like 310,000, and that of Sunderland under 105,000. You will see from the report I send by the same post that our workpeople's ordinary subscriptions last year, in spite of unprecedented depression in trade, reached £2,469 0s. 5d., and that an additional £400 was raised by a Workmen's Industrial Exhibition. These figures speak for themselves, and in justice to my committee and the workpeople in general, I will be obliged if you would insert this in your next issue.

"A. W. Webster, Chairman,
"Workmen's Governors, Sunderland Infirmary."

